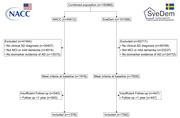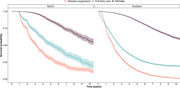# Real‐world effectiveness of anti‐amyloid therapies: the feasibility of obtaining historic controls from large‐scale disease registries

**DOI:** 10.1002/alz.087855

**Published:** 2025-01-09

**Authors:** Linus Jönsson, Bengt Winblad, Anders Wimo, William L Herring

**Affiliations:** ^1^ Karolinska Institutet, Solna, Stockholm Sweden; ^2^ Karolinska Institutet, Stockholm Sweden; ^3^ Karolinska Institutet, Solna Sweden; ^4^ Karolinska Institute, Stockholm, Södermanland and Uppland Sweden

## Abstract

**Background:**

Novel anti‐amyloid therapies (AAT) for Alzheimer’s Disease (AD) have recently been approved in the United States, Japan and China, and are under regulatory review in Europe. Questions remain regarding the long‐term effectiveness and value of these drugs when used in routine clinical practice. Data from follow‐up studies will be important to inform their optimal use, including criteria for treatment initiation, monitoring strategies, stopping rules, pricing and reimbursement considerations. Newly initiated treatment registries will only or mainly enroll patients receiving AAT with no control arm ‐ for comparisons with of health outcomes in untreated patients, external historic control data will be required.

**Method:**

We reviewed the literature to identify disease registries with relevant design and inclusion criteria.Patient‐level data was obtained from two registries deemed to be promising as sources of historic control data: the Swedish Registry for Cognitive Disorders (SveDem) and the National Alzheimer’s Coordinating Centers (NACC). From these registries we selected patients fulfilling key criteria for treatment with AAT and analyzed longitudinal data on relevant endpoints (disease progression, functional status and mortality) using survival analysis methods. Sample size requirements for evaluating the effect of AAT on these endpoints were estimated using the Lachin & Foulkes method assuming a risk ratio of 0.7 with treatment.

**Result:**

35 disease registries were identified from 15 countries. Many did not include the relevant patient populations, endpoints or had insufficient follow‐up. From SveDem and NACC, 1378 and 7392 patients respectively were included, respectively. The median time to disease progression was 1.9‐3.2 years, time to full‐time care 2.7‐5.3 years, and median overall survival 11.4‐13.6 years. Approximately 248 events would be required to detect a difference in time to event with 80% power. In SveDem, a total number of 2717 disease progression events, 3916 full‐time care events and 842 mortality events were observed.

**Conclusion:**

Follow‐up studies will be of key importance for confirming the clinical effectiveness and economic value of DMT in routine care. Established registry infrastructures with available historic datasets will play an important role. International collaboration may be needed to achieve sufficient sample size for assessment effects on some endpoints, such as overall survival.